# Pregnant woman awareness of obstetric danger signs in developing country: systematic review

**DOI:** 10.1186/s12884-023-05674-7

**Published:** 2023-05-16

**Authors:** Esti Yunitasari, Filomena Matos, Hakim Zulkarnain, Dewi Indah Kumalasari, Tiyas Kusumaningrum, Tantya Edipeni Putri, Ah. Yusuf, Nining Puji Astuti

**Affiliations:** 1 Faculty of Nursing, Universitas Airlangga, Surabaya, Indonesia; 2 Escola Superior de Saúde, University of Algarve, Faro, Portugal; 3 UICISA:E, Health Sciences Research Unit: Nursing, Coimbra, Portugal; 4 Department of Nursing, Faculty of Medicine and Health Science, Satya Wacana Christian University, Salatiga, Indonesia; 5 Student of Medical and Surgical Nursing Specialist Program, Faculty of Nursing, Universitas Airlangga, Surabaya, Indonesia

**Keywords:** Awareness, Maternal health, Maternal mortality, Obstetric danger signs, Health seeking action

## Abstract

**Background:**

Mother's awareness of obstetric danger signs is the degree of a pregnant woman to fully utilize her knowledge of the signs and symptoms of complications of pregnancy, which helps the mother and family to seek medical help immediately. High maternal and infant mortality rates in developing countries are due to a combination of a lack of quality, resources and access to health services coupled with mother’s lack of awareness. The purpose of this study was to collect current empirical studies to describe the pregnant women awareness about the obstetric danger sign in developing country.

**Method:**

This review employed the Prisma-ScR checklist. The articles searched in four electronic databases (Scopus, CINAHL, Science Direct, Google Scholar). Variables that used to search the articles (pregnant woman, knowledge, awareness, danger signs pregnancy). The Framework used to review is PICOS.

**Result:**

The results of the article found 20 studies which met inclusion criteria. The determinants were high educational status, more pregnancy experience, more ANC visit, and labour in the health facility.

**Conclusion:**

The level of awareness is low to medium, only some have fair awareness, in which related to determinant. The recommended effective strategy is to improve the ANC program by assess the risk of obstetric danger sign promptly, assess the barrier of health seeking related to the family support, i.e. the husband and the elderly. Additionally, use MCH handbook or mobile application to record the ANC visit and communicate with the family.

## Introduction

Maternal Mortality Rate (MMR) is one indicator of women's health of Goals 3 Sustainable Development Goals [1, 2]. It is estimated that low- and middle-income countries contributed to the 56% - 99% of total global MMR (Mother Mortality Rate) [3, 4]. In order to design an effective intervention for to reduce MMR in developing country, obstetric danger sign awareness level is a necessary factor to address [5]. Mother's awareness of obstetric danger signs in developing country is relatively low regardless many local and national program attempt [1, 6–8]. It is crucial to increase pregnant women knowledge of obstetric danger signs through education and awareness-raising efforts [9–12]. Inseparable with efforts to increase knowledge, it is necessary to understand how different community utilize the of health care facility according to the awareness level [4]. Low ANC visit in the health care facility affected the level of awareness to obstetric danger sign which then add the number of MMR [1, 13–15].

Mother's awareness of obstetric danger signs is integral of knowledge, which refers to the extent a pregnant woman is able to utilize her knowledge of the signs and symptoms of potential complications during pregnancy, childbirth, and the postpartum period [4, 5, 16, 17]. With this information, she is better equipped to anticipate possible issues and seek timely medical attention, which can reduce the risk of maternal and neonatal morbidity and mortality 14,18. Recent studies highlight the importance of maternal awareness of obstetric danger signs in which the more aware women act differently to unaware women. Aware woman in Nigeria were more likely to seek skilled birth attendance [18]. Similarly, aware woman in Cameroon would more likely to seek antenatal care, give birth in a health facility, and had better maternal health outcomes [19]. Another study in Ethiopia found that these women were keen to seek postnatal care within the recommendation time [4, 7, 8, 19–23].

Mother and family should immediately seek medical help if the obstetric danger sign is present [21, 24, 25]. There is significant variation in the statistics of mother seeking help for obstetric danger signs by utilizing ANC visit (Antenatal Care) a minimum of four visit during the pregnancy period [1]. Some developing countries reported as follows: Indian women were 72% [5], Tanzanian women were 64.7% [3], Indonesian women were 96,9% [1], Ethiopian ranged from around 29.1% [4] to around 39.08% [26]. This variation of statistics highlights the need to improve maternal education effectiveness and healthcare access in developing countries [5]. Because women who aware about the obstetric danger sign are 3,47 times more likely to utilize the ANC care and reduce maternal and neonatal mortality rates [17].

The lack of knowledge about sign and symptoms leads to low awareness, then mismanagement of the complications in pregnancy, childbirth, postpartum [24]. Additionally, some mother in developing country reports the needs to consult with family to access the health care facility. Coupled with the lack of drugs and equipment, the unavailability, and the long queue of health service [27]. The combination of lack of quality, resources and access to health services coupled with mother’s low awareness and family factors become the barrier for maternal wellbeing [28, 29]. The purpose of this study was to collect current empirical studies to describe the pregnant women awareness about the obstetric danger sign in developing country.

## Method

This review employed the PRISMA-ScR checklist. This review sought to answer these questions:

1. What is the current empirical data of pregnant mother awareness about the obstetric danger sign in developing country?
2. What are the determinant of awareness?
3. What is the effective strategy to improve awareness for developing country?

This review designed to pool all relevant literature which emphasizes the topic of woman knowledge of pregnancy danger sign. Broad keywords used to find as many relevant results as possible.

The research explored must contain the following inclusion criteria: a) research involving pregnant women of all ages and parity, b) conducted in developing countries, c) quantitative research methods, d) cross sectional research design, and e) employed more than 100 respondents.

The found research was directly excluded if a) Published 2017 - 2022, b) research in languages other than English and Bahasa, c) research that discusses these factors but from the results of the intervention, d) community service reports that provide treatment for the factors, and e) research conducted in the developed country (based on the World Bank Country and Lending Groups).

Electronic databases used were Scopus, Science Direct, CINAHL, and Google Scholar. Articles known by the researcher and not collected during the search process on the database added manually. The article search conducted in June 2022. The search term based on the PICO framework which is Patient: (*Mother OR Woman)*, Interest: *(Knowledge) AND (Awareness), AND (danger signs of pregnancy OR danger signs during pregnancy),* there were no Comparison and Outcome necessary to highlighted. The details of search strategy shown in the (Table 1). To increase the search accuracy, the bibliography of related articles screened as well.

**Table 1:** PICO Systematic Review Keywords

PICO		
	***Patient/Problem***	*(Mother OR Woman)*
	***Interest/Intervention***	*(Knowledge)* *AND* *(Awareness)* *AND* *(danger signs of pregnancy OR danger signs during pregnancy)*
**Database**	**Operational Keywords**	**Limitation**
**Scopus**	mother **OR** woman **AND** knowledge **AND** awareness **AND** danger **AND** signs **AND** of **AND** pregnancy **OR** danger **AND** signs **AND** during **AND** pregnancy	**Publication Year:** 2015 – 2022
**Science Direct**	Mother **OR** Woman **AND** Knowledge **AND** Awareness **AND** danger signs of pregnancy **OR** danger signs during pregnancy	**Publication Year:** 2015 – 2022 **Article Type:** Research Article **Subject Areas:** Nursing and Health Professions
**CINAHL**	Mother **OR** Woman **AND** Knowledge **AND** Awareness **AND** danger signs of pregnancy **OR** danger signs during pregnancy	**Limit to:** Full Text **Publication Year:** 2015 – 2022 **Language:** English **Geography:** Mexico & Central/ South America, Asia, Africa, Middle East

Three authors responsible to evaluate the pooled literature. First, they removed the duplicates, then continue to the screening phase. Second, they used the filter and exclusion criteria by reading the title and the abstract. Third, read all the full texts and apply the inclusion criteria. Lastly the articles that pass until this stage appraised critically using the CASP checklist [30]. The articles that judged to be of high quality included in the review for systematic qualitative analysis.

Data extraction included the design, sample, variable, instrument, analysis, and result. The narrative literature review built thematically. The most common theme that emerge in all articles collected and reported in a way that will rhyme with the topic and the review questions.

## Result

### Pooled articles and the characteristics

**Fig. 1:**
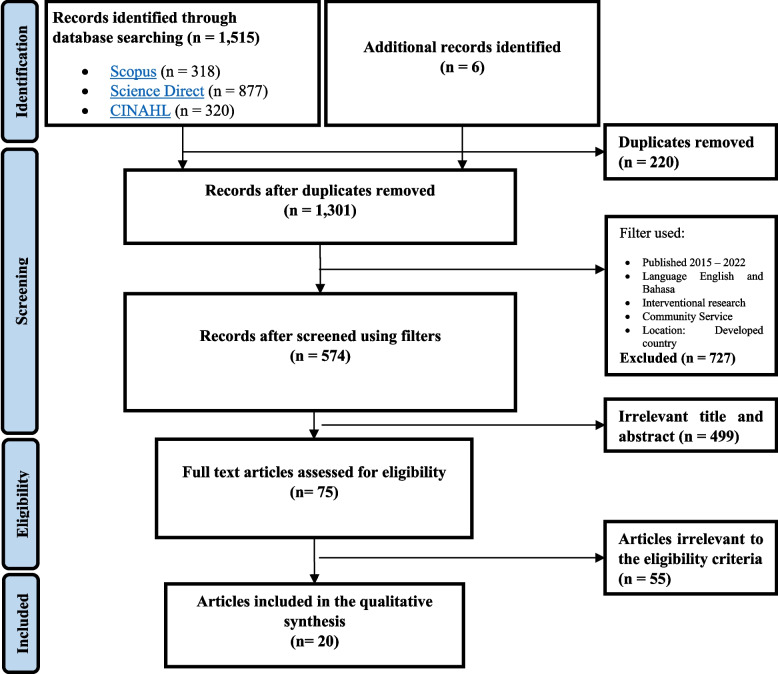
PRISMA Diagram

One thousand five hundred fifteen articles collected on the first literature search and 6 articles added from manual search. 220 articles removed due to duplication. 727 articles excluded according to the filters. 499 articles removed due to irrelevant title and abstract. Seventy-five full paper articles read and applied the eligibility criteria which resulted on 20 articles included in the review (Fig. 1 for PRISMA flow diagram). The references of all the included articles reviewed to find additional sources, yet the team found no eligible article.

The overview of key characteristics found in the 20 included articles described in the Table 2. All studies conducted in the developing countries, mostly from Africa, namely Cameroon, Ethiopia, Madagascar, Nigeria, Republic of Congo, and, Tanzania, the rest was from Asia, which are Bhutan, India, Indonesia, and Papua New Guinea. All the study was a cross sectional study in which some of them was community based and the rest was clinic or hospital based. All the study were measuring the level of knowledge of mother about obstetric danger sign in which consist of pregnancy, childbirth, and postpartum danger sign. Only one study includes additional newborn danger sign [31].

**Table 2:** Overview of key characteristics of pooled articles

No	First Author	Year of Publication	Location/ Country	Topic	Total respondents	Education level	Economic status	Knowledge level	Definition of knowledgeable level	Most known danger sign by respondents	Recommendation to increase awareness	Source of information
1	Vijay et al. (2015) [24]	2015	OPD of Lata Mangeshkar Hospital, Nagpur, **India**	Pregnant woman danger sign	100 pregnant women attending ANC			1. 6,38% were having good knowledge 2. 20% have fair knowledge 3. 73% have poor knowledge	1. > 15 Good knowledge (> 75%) 2. 10 – 15 fair knowledge (50–75%) 3. < 10 poor knowledge (< 50%)	During pregnancy danger 1. Bleeding 50% 2. Swollen hands and face 48% 3. Blurred vision 35% 4. Lack of blood 22% Delivery danger 1. Severe vaginal 50% 2. Retained placenta 23% 3. Labour lasting > 12 h 16% Postpartum danger 1. Severe bleeding 50% 2. Swollen hand, feet, face 48% 3. Visual disturbances 35% Weakness 22%	1. A well-planned strategy for danger sign education	1. Health personnel 33% 2. Mass media 67%
2	Belay & Limenih (2020) [32]	2020	Community of Farta Woreda, Northwest **Ethiopia**	Obstetric danger sign	735 mothers delivered baby at least once in the last 2 years			1. Danger sign during pregnancy Knowledgeable 71,4% Not 28,6% 2. Danger sign during childbirth Knowledgeable 65,3% Not 34,7% 3. Danger sign during postpartum Knowledgeable 77% Not 23%	At least mentioned 3 danger signs considered as knowledgeable	Obstetric danger 1. Vaginal bleeding 48.4% 2. Swollen hands and face 29.1% Delivery danger 1. Severe vaginal bleeding 61.1% 2. Malpresentation (abnormal position of the fetus) 36.2% Postpartum danger 1. Vaginal bleeding 72.2% Severe headache 28.8%	1. Increasing antenatal- care coverage 2. Educating women 3. Increasing institutional delivery	1. Health personnel 62,4% 2. Friends 18,8% 3. Mass Media 7,8%
3	Wassihun et al. (2020) [21]	2020	Community of Shashamane town, Oromia region, **Ethiopia**	Obstetric danger sign	422 recently delivered mother (< 12 months)			1. 40.5% were having good knowledge 2. 59.5% were having poor knowledge	At least spontaneously mentioned 2 danger signs considered as knowledgeable Less than that considered as poor	Obstetric danger 1. vaginal bleeding (64.7%) 2. absent or decreased fetal movements (38.6%) Childbirth danger 1. Bleeding (60%) 2. Absent or decrease fetal movements (28.4%) Postpartum danger 1. Postnatal bleeding (63.3%) Postnatal fever (38.9%)	1. Mobilizing communities 2. Encouraging pregnant women to attend antenatal clinics 3. Providing health information dis- semination	
4	Mardiyanti et al. (2019) [13]	2019	Community of Surabaya City, **Indonesia**	Pregnant women danger sign	125 pregnant women			1. 72,4% have good knowledge 2. 26,9% have less knowledge	Not explained			
5	Bogale & Markos (2015) [22]	2015	Community of Goba district, **Ethiopia**	Obstetric Complication danger sign	562 recently delivered mother (< 12 months)			1. Danger sign during pregnancy Knowledgeable 31,9% Not 68,1% 2. Danger sign during childbirth Knowledgeable 27% Not 73% 3. Danger sign during postpartum Knowledgeable 22,1% Not 77,9%	At least spontaneously mentioned 3 danger signs considered as knowledgeable	During pregnancy 1. Severe vaginal bleeding 71,3% 2. Severe headache 53,7% Childbirth danger 1. Vaginal bleeding 29% 2. Prolonged labour (> 12 h) 27% 3. Retained placenta 23% Postpartum danger 1. Severe vaginal bleeding 76,5% Severe headache 39,8%	1. Every woman should be made aware of complications during pregnancy, childbirth/ labour and the postpartum periods 2. Interventions targeting improvement of maternal health need including the quality of information offered to pregnant women during ANC follow up is recommended	
6	Tjandraprawira & Ghozali (2019) [1]	2019	Patient of maternity ward, Majalengka General District Hospital, **Indonesia**	Pregnancy danger sign	127 recently delivered postpartum women treated in the maternity ward			1. 61,4% have good knowledge 2. 38,6% have less knowledge	Scored above average score (25.41 (± 3.44)) considered as knowledgeable	During pregnancy 1. Swellings of the hands, feet and face 78% 2.‘Water break’ or premature rupture of membrane (PROM) 85% 3. Bleeding early in pregnancy 93.7% 4. Prolonged coughing 94% 5. Episode of palpitations during pregnancy 76% 6. Repeated episodes of diarrhea needed an urgent referral to a physician 93% During Childbirth 7. Bleeding during labor 83.5% 8. Seizure 90% Postpartum 9. Prolonged anxiety required a consultation with a physician 95% 10. Prolonged sadness after delivery (postpartum depression) is normal 29% 11. Women knew that only fevers more than 2 days during the postpartum/puerperium period required referrals to the nearest health facility 29% 12. Patients had known that foul-smelling vaginal discharge during puerperium was an ominous sign 79.5%	1. MCH (Mother and Child Health) book	
7	Vallely et al. (2019) [16]	2019	Community of Hiri District (Central Province), Karkar Madang Province), and Asaro (Eastern Highlands Province), **Papua New Guinea**	Pregnancy danger sign	482 recently delivered women (1 – 2 years prior) attending ANC clinic			1. Any Danger sign during pregnancy (183/459) Knowledgeable 39,9% Not 60% 2. ≥ 3 danger signs during pregnancy (39/183) Knowledgeable 21,3% Not	1. Mentioned any danger sign 2. Mentioned ≥ 3 danger signs	1. Fever 47.5% 2. Bleeding in pregnancy 39.3% 3. Swelling of the legs/ arms 36.6% 4. Headaches and dizziness 27.9%	1. Health care worker harness the opportunity of the antenatal clinic to provide women with information on the importance of recognising danger signs during pregnancy 2. The importance to seek professional, skilled care promptly should problems arise	1. Health personnel 53.6%
8	Woldeamanuel et al. (2019) [25]	2019	Community of Angolela Tera District, Northern **Ethiopia**	Obstetric Complication danger sign	563 pregnant women			1. Danger sign during pregnancy Knowledgeable 56,1% Not 2. Danger sign during childbirth Knowledgeable 58,8% Not 3. Danger sign during postpartum Knowledgeable 34,5% Not	At least spontaneously mentioned 3 danger signs considered as knowledgeable	1. Excessive vaginal bleeding 72,6% 2. Swollen hands 12,8% 3. Convulsion 9,8% 4. Reduced fetal movement 54% 5. High fever 43,9%		1. Health care workers 60% 2. Neighbors 34.3% 3. Media 5.7%
9	Bililign & Mulatu (2017) [20]	2017	Community of Raya Kobo district of **Ethiopia**	Obstetric danger signs	493 recently delivered mother (< 12 months)			1. Danger sign during pregnancy Knowledgeable 46,7% Not 53,3% 2. Danger sign during childbirth Knowledgeable 27,8% Not 72,2% 3. Danger sign during postpartum Knowledgeable 26,4% Not 73,6%	At least spontaneously mentioned 3 danger signs considered as knowledgeable	Obstetric danger 1. Vaginal bleeding 83,5% 2. Accelerated/ decreased fetal movement 38,1% Childbirth danger 1. Vaginal bleeding 91,2% 2. Retained placenta 58,7% Postpartum danger 1. Vaginal bleeding 89,2% 2. Offensive vaginal discharge 23,3% Severe headache 23,1%	1. Empowering women 2. Improving the quality of health information about danger signs during ANC follow up 3. Promoting institutional delivery are the recommended interventions	
10	Amenu et al. (2016) [7]	2016	Patient of Mechekel District Health Centers, East Gojjam Zone, Northwest **Ethiopia**	Obstetric danger signs	411 Postnatal mothers / recently delivered / postpartum women treated in the health centre			1. Danger sign during pregnancy Knowledgeable 52,1% Not 2. Danger sign during childbirth Knowledgeable 53,3% Not 3. Danger sign during postpartum Knowledgeable 46,4% Not	If scored above the calculated mean is considered knowledgeable	During pregnancy danger 1. Vaginal bleeding 53,8% 2. Severe headache 52,3% 3. Increased/ decreased fetal movement 43,5% Childbirth danger 1. Vaginal bleeding 56,8% 2. Prolonged labour (> 12 h) 46,9% 3. Retained placenta 44,4% Postpartum danger 1. Vaginal bleeding 60,2% 2. Foul smell of vaginal discharge 38,5% High fever with or without abdominal pain 36,8%		1. Health service providers 76.3% 2. Mass media 20.5% 3. Friends 24.0% 4. Community 10.9%
11	Maseresha et al. (2016) [4]	2016	Community of Erer district, Somali region, **Ethiopia**	Obstetric danger sign	666 pregnant women			1. Danger sign during pregnancy Knowledgeable 25,5% Not 2. Danger sign during childbirth Knowledgeable 31,8% Not 3. Danger sign during postpartum Knowledgeable 19,1% Not	At least spontaneously mentioned 2 danger signs considered as knowledgeable	During pregnancy danger 1. Vaginal bleeding 25% Childbirth danger 1. Prolonged labour 26% 2. Excessive bleeding 15% Postpartum danger 1. Excessive vaginal bleeding 20% 2. Abdominal pain 10% Fever 8%	1. Increasing ANC service utilization would improve pregnant women’s knowledge about obstetric danger signs and symptoms	
12	Salem et al. (2018) [31]	2018	Community of Ambanja, **Madagascar**	Obstetric complications danger sign and newborn danger sign	372 recently delivered mother (< 12 months)			1. Danger sign during pregnancy Knowledgeable 80,9% Not 2. Danger sign during childbirth Knowledgeable 51,9% Not 3. Danger sign during postpartum Knowledgeable 50,8% Not 4. Danger sign of newborn Knowledgeable 53,2% Not	At least spontaneously mentioned 1 danger signs considered as knowledgeable	During pregnancy danger 1. Fever 41,1% 2. Headache 32,0% 3. Swollen hands and body 28.8% 4. Vaginal bleeding 26.9% Childbirth danger Postpartum danger	1. Mobile health (mHealth) as the remote ANC solution	
13	Nigussie et al. (2019) [8]	2019	Community of Bahir Dar city administration, North West, **Ethiopia**	Obstetric danger sign	701 recently delivered mother (< 12 months)			1. 37,9% have good knowledge 2. have less knowledge	At least mentioned 2 danger signs considered knowledgeable	During pregnancy danger 1. Severe vaginal bleeding 81,6% 2. Loss of consciousness 34,1% 3. Increase/ decrease of fetal movement 32,7% 4. Difficulty in breathing 30,5% Childbirth danger 1. Severe vaginal bleeding 82,2% 2. Retained placenta 59,6% 3. Prolonged labour 51,6% 4. Loss of consciousness 32,7% Postpartum danger 1. Severe vaginal bleeding 85,3% 2. Loss of consciousness 32,7% Swelling of face/ hands 28,5%	1. Provision of information on ODSs during the ANC period to facilitate the recognition on ODSs 2. Improve access to skilled attendance services	
14	Dangura (2020) [23]	2020	Community of Dale district, Southern **Ethiopia**	Obstetric danger sign	782 recently delivered mother (< 12 months)			1. Danger sign during childbirth Knowledgeable 45,5% Not 2. Danger sign during postpartum Knowledgeable 29,1% Not	At least mentioned 2 danger signs considered knowledgeable	Childbirth danger 1. Severe vaginal bleeding 68.4% 2. Severe headache 29.4% 3. Convulsion 27.5% 4. High fever 24.6% 5. Loss of consciousness 19.7% 6. Labor lasting greater than 12 h 17.0% 7. Placenta not delivered 30 min after delivery 18.7% Postpartum danger 1. Severe vaginal bleeding 16% 2. Severe headache 8.6% 3. Convulsion 7% 4. Swollen hand or face 5.3% 5. High fever 6.8% 6. Loss of consciousness 6.2% 7. Difficult of breathing 6.6% 8. Severe weakness 7% 9. Malodorous vaginal discharge 8.9%		
15	Mwilike, Nalwadda, et al. (2018) [33]	2018	Patient of two health centers in Kinondoni Municipality, Dar es Salaam, **Tanzania**	Obstetric danger sign	384 postpartum women who were seeking immunization services for their children			Danger sign during pregnancy 1. Knowledgeable (≥ 4) 31% 2. Low knowledge (1–3) 57,8% 3. Not (none) 2,7%	Mentioned ≥ 4 danger signs is having sufficient knowledge	1. Vaginal bleeding 81.2% 2. Edema 46.3% 3. Headache 43.6%		1. RCHC (Reproductive and Child Health Clinic) 81.8% 2. Social gatherings 17.4% 3. Radio 0.8%
16	Nkamba et al. (2021) [6]	2021	Woman visited health facility for ANC **Democratic Republic of Congo**	Obstetric danger sign	4512 recently delivered women attending ANC clinic			Obstetric danger sign 1. 23% Good knowledge 2. 76% Poor knowledge	Mentioned more than median (> 2)	During pregnancy danger 1. Vaginal bleeding 18% 2. Headache or blurred vision 2% 3. Swollen face or hands 18% 4. Reduced or no fetal movement 18% 5. Tiredness or breathlessness 8% 6. Cough or difficulty breathing 5% 7. Fever 18% 8. Convulsion (Not Mentioned)		
17	Emeh et al. (2021) [19]	2021	Woman delivered in the Buea Regional Hospital **Cameroon**	Obstetric danger sign	532 woman 24 h postpartum			Obstetric danger sign 73,3% knowledgeable / aware		During pregnancy 1. Severe vaginal bleeding 71,4% 2. Convulsion/ loss of consciousness 35,7% 3. Severe headache or blurred vision 41,7% 4. Reduced fetal movement 60,9% 5. Swollen face or hands 18% During Labor 6.Prolonged labor (> 12 h) 18,7% 7. Retained placenta 17,3% 8. Fast or difficulty in breathing 39,8% During Postpartum 9. Fever 62% 10. Severe vaginal bleeding 11. Foul-smelling vaginal discharge		
18	Shamanewadi et al. (2020) [5]	2020	Pregnant women (18 – 32 y.o) attended ANC in the Primary Health Centre, Nandagudi, Bengaluru rural **India**	Obstetric danger sign	210 women attending the ANC clinic	1.1.55% high school 2.2.9% illiterate 3.3.6% higher education	80% lower – middle class	Obstetric danger sign 100% knowledgeable	Mentioned at least 3 danger signs	1. Fever 37.1% 2. Bleeding PV 100% 3. Reduced fetal movements 0.95% 4. Leaking PV 1.90% 5. Blurred vision 3.33% 6. Abdomen pain 100% 7. Loss of consciousness 0.95% 8. Convulsions 100%	Screening for high risk conditions – a free managerial tool during the ANC	1. ANC clinic in PHC 2. Health care staff
19	Oguntunde et al. (2021) [18]	2021	Nigeria	Obstetric danger sign	1624 < 25 years old women, join the The Nigeria Maternal Newborn and Child Health Programme (MNCH2), a UK Aid funded 5-year (2014–2019)			1. Danger sign during pregnancy Knowledgeable 50% 2. Danger sign during childbirth Knowledgeable 58% 3. Danger sign during postpartum Knowledgeable 41,45%	Mentioned at least 2 danger sign		Solution on the need of always ask Husband permission before seeking care although danger sign appear	
20	Tamang et al. (2021) [14]	2021	Pregnant women aged ≥ 18 years and attending the ANC clinic at Gyaltsuen Jetsun Pema Mother and Child Hospital **Bhutan**	Obstetric danger sign	422 women attending the ANC clinic			Obstetric danger sign 1. 4.7% good knowledge 2. 58.1% satisfactory knowledge 3. 37.2% poor knowledge	Good = Scored ≥ 80% Satisfactory = Scored 60 – 79% Poor = < 60%	1. Pulmonary embolism 1,70% 2. Preterm labour 7,70% 3. Reduced Foetal movement 14,90% 4. Fever 23,80% 5. Preeclampsia 24,80% 6. Hyperemesis 34,30% 7. Vaginal bleeding 67,70%	Use the MCH handbook	1. Nurse & Midwife 77,00% 2. Family & relatives 58,50% 3. Media 54,30% 4. MCH Handbook 49,20% 5. Doctor 19,40% 6. Others 17,30%

The sum of respondents from all articles were around 13,443 women. The respondents characteristics were pregnant woman [4, 5, 13, 14, 18, 24, 25] mother delivered in the last 1 - 2 years [16, 32], recently delivered mother (<12 months) [1, 6–8, 19–23, 31], and one study did not clearly state the time period from the last labour [33]. Meanwhile, the place of respondents recruited were in the community or in health facilities. Some of the respondents in health facilities currently hospitalized after giving birth, some visited ANC and the last one was looking for immunizations for their children.

### Operation definition of the knowledge level

Details of the level of knowledge report can be seen in the Table 3. Eight articles report the three knowledge levels of obstetric danger sign [4, 7, 18, 20, 22, 23, 25, 31, 32], while the rest reported two or had summarized the three categories. The method used to measure the level of knowledge was by ask the respondents to mention obstetric danger sign. If they can mention a certain number of danger sign above the benchmark, then those respondents rated as knowledgeable or have good knowledge. While the benchmark values used are slightly different. The majority of studies use the number of danger signs, namely ≥ 4 dangers [33], ≥ 3 obstetric danger signs [5, 16, 20, 22, 25, 32], ≥ 2 danger sign [4, 8, 18, 21, 23], dan ≥ 1 danger sign [31]. Three other studies used different measurement methods, two studies used the mean as the limit of assessment [1, 6, 7, 19], and two study used a percentage of more than 75% [14, 24].

**Table 3:** Operation definition of level of knowledge

No	First Author	Knowledge level	Definition of knowledgeable level
		≥ 4 danger signs	
1	Mwilike et al. (2018) [27, 33]	Danger sign during pregnancy 1. Knowledgeable (≥ 4) 31% 2. Low knowledge (1–3) 57,8% 3. Not (none) 2,7%	Mentioned ≥ 4 danger signs is having sufficient knowledge
		≥ 3 danger signs	
2	Vallely et al. (2019) [16]	1. Any Danger sign during pregnancy (183/459) Knowledgeable 39,9% Not 60% 2. ≥ 3 danger signs during pregnancy (39/183) Knowledgeable 21,3% Not -	1. Mentioned any danger sign 2. Mentioned ≥ 3 danger signs
3	Belay & Limenih (2020) [32]	1. Danger sign during pregnancy Knowledgeable 71,4% Not 28,6% 2. Danger sign during childbirth Knowledgeable 65,3% Not 34,7% 3. Danger sign during postpartum Knowledgeable 77% Not 23%	At least mentioned 3 danger signs considered as knowledgeable
4	Bogale & Markos (2015) [22]	1. Danger sign during pregnancy Knowledgeable 31,9% Not 68,1% 2. Danger sign during childbirth Knowledgeable 27% Not 73% 3. Danger sign during postpartum Knowledgeable 22,1% Not 77,9%	At least spontaneously mentioned 3 danger signs considered as knowledgeable
5	Woldeamanuel et al. (2019) [25]	1. Danger sign during pregnancy Knowledgeable 56,1% Not 2. Danger sign during childbirth Knowledgeable 58,8% Not 3. Danger sign during postpartum Knowledgeable 34,5% Not	At least spontaneously mentioned 3 danger signs considered as knowledgeable
6	Bililign & Mulatu (2017) [20]	1. Danger sign during pregnancy Knowledgeable 46,7% Not 53,3% 2. Danger sign during childbirth Knowledgeable 27,8% Not 72,2% 3. Danger sign during postpartum Knowledgeable 26,4% Not 73,6%	At least spontaneously mentioned 3 danger signs considered as knowledgeable
7	Shamanewadi et al. (2020) [5]	100% knowledgeable	Mentioned at least 3 danger signs
		≥ 2 danger signs	
8	Maseresha et al. (2016) [4]	1. Danger sign during pregnancy Knowledgeable 25,5% Not 2. Danger sign during childbirth Knowledgeable 31,8% Not 3. Danger sign during postpartum Knowledgeable 19,1% Not -	At least spontaneously mentioned 2 danger signs considered as knowledgeable
9	Dangura (2020) [23]	1. Danger sign during childbirth Knowledgeable 45,5% Not 2. Danger sign during postpartum Knowledgeable 29,1% Not	At least mentioned 2 danger signs considered knowledgeable
10	Nigussie et al. (2019) [8]	1. 37,9% have good knowledge 2. have less knowledge	At least mentioned 2 danger signs considered knowledgeable
11	Wassihun et al. (2020) [21]	1. 40.5% have good knowledge 2. 59.5% have poor knowledge	At least spontaneously mentioned 2 danger signs considered as knowledgeable
12	Nkamba et al. (2021) [6]	Obstetric danger sign 1. 1. 76% Poor knowledge 2. 23% Good knowledge	Mentioned more than median (> 2)
13	Oguntunde et al. (2021) [18]	1. Danger sign during pregnancy Knowledgeable 50% 2. Danger sign during childbirth Knowledgeable 58% 3. Danger sign during postpartum Knowledgeable 41,45%	Mentioned at least 2 danger sign
		≥ 1 danger signs	
14	Salem et al. (2018) [31]	1. Danger sign during pregnancy Knowledgeable 80,9% Not 2. Danger sign during childbirth Knowledgeable 51,9% Not 3. Danger sign during postpartum Knowledgeable 50,8% Not 4. Danger sign of newborn Knowledgeable 53,2% Not	At least spontaneously mentioned 1 danger signs considered as knowledgeable
		Scored above mean	
15	Tjandraprawira & Ghozali (2019) [1]	1. 61,4% have good knowledge 2. 38,6% have less knowledge	Scored above average score (25.41 (± 3.44)) considered as knowledgeable
16	Amenu et al. (2016) [7]	1. Danger sign during pregnancy Knowledgeable 52,1% Not 2. Danger sign during childbirth Knowledgeable 53,3% Not 3. Danger sign during postpartum Knowledgeable 46,4% Not	If scored above the calculated mean is considered knowledgeable
		Benchmark > 75%	
17	Vijay et al. (2015) [24]	1. 6,38% were having good knowledge 2. 20% have fair knowledge 3. 73% have poor knowledge	1. > 15 Good knowledge (> 75%) 2. 10 – 15 fair knowledge (50–75%) 3. < 10 poor knowledge (< 50%)
18	Tamang et al. (2021) [14]	Obstetric danger sign 1. 4.7% good knowledge 2. 58.1% satisfactory knowledge 3. 37.2% poor knowledge	1. Good = Scored ≥ 80% 2. Satisfactory = Scored 60 – 79% 3. Poor = < 60%
		Not explained	
19	Mardiyanti et al. (2019) [13]	1. 72,4% have good knowledge 2. 26,9% have less knowledge	Not explained
20	Emeh et al. (2021) [19]	73,3% knowledgeable / aware	Not explained

### The mother level of awareness according to the knowledge level

Because the categories used by each study to describe the level of knowledge are different, the authors will report based on the operational definitions used. Studies using the understand 4 danger signs measure have a good knowledge level of 31% and the remaining 69% have low or no knowledge at all [33]. Next, using the 3 danger sign measure, the highest percentage of the good knowledge category is 71.4% regarding the danger sign during pregnancy [32] and the lowest is 22.1% [22] about the danger sign during postpartum. Additionally 100% knowledgeable found in the overall danger sign [5].

In this measurement category there are many reported knowledge level values, for more details see Table 4. The next measurement is 2 danger signs, the highest value is during childbirth at 45.5% while the lowest is 19.1% during postpartum [4]. The measurement of 1 danger signs tends to be high at 80.9% during pregnancy and the lowest is 50.8% during postpartum [31]. For other categories, the highest level of knowledgeable is around 50-60% [1, 7, 24]. The other one studies which did not mention the operational definition of level of knowledge are reported as it is [19].

**Table 4:** Mother level of knowledge of obstetric danger sign

No	Author	Definition of knowledgeable level	Danger sign									
			During pregnancy		During childbirth		During post-partum		Newborn		Overall	
			Good	Low – Not	Good	Low – Not	Good	Low – Not	Good	Low – Not	Good	Low – Not
1	Mwilike et al. (2018) [27, 33]	≥ 4 danger signs	31%	69%	-	-	-	-	-	-	-	-
2	Vallely et al. (2019) [16]	≥ 3 danger signs	21,3%	78,7%	-	-	-	-	-	-	-	-
3	Belay & Limenih (2020) [32]		71,4%	28,6%	65,3%	34,7%	22,1%	77,9%	-	-	-	-
4	Bogale & Markos (2015) [22]		31,9%	68,1%	27%	73%	22,1%	77,9%	-	-	-	-
5	Woldeamanuel et al. (2019) [25]		56,1%	-	58,8%	-	34,5%	-	-	-	-	-
6	Bililign & Mulatu (2017) [20]		46,7%	53,3%	27,8%	72,2%	26,4%	73,6%	-	-	-	-
7	Shamanewadi et al. (2020) [5]		-	-	-	-	-	-	-	-	100%	-
8	Maseresha et al. (2016) [4]	≥ 2 danger signs	25,5%	-	31,8%	-	19,1%	-	-	-	-	-
9	Dangura (2020) [23]		-	-	45,5%	-	29,1%	-	-	-	-	-
10	Nigussie et al. (2019) [8]		-	-	-	-	-	-	-	-	37,9%	-
11	Wassihun et al. (2020) [21]		-	-	-	-	-	-	-	-	40,5%	59,5%
12	Nkamba et al. (2021) [6]		-	-	-	-	-	-	-	-	76%	23%
13	Oguntunde et al. (2021) [18]		50%	-	58%	-	29,1%	-	-	-	-	-
14	Salem et al. (2018) [31]	≥ 1 danger signs	80,9%	-	51,9%	-	50,8%	-	53,2%	-	-	-
15	Tjandraprawira & Ghozali (2019) [1]	Scored above mean	-	-	-	-	-	-	-	-	61,4%	38,6%
16	Amenu et al. (2016) [7]		52,1%	-	53,3%	-	46,4%	-	-	-	-	-
17	Vijay et al. (2015) [24]	Understand > 75%	-	-	-	-	-	-	-	-	6,38%	93%
18	Tamang et al. (2021) [14]		-	-	-	-	-	-	-	-	62,8%	37,2%
19	Mardiyanti et al. (2019) [13]	Not explained	-	-	-	-	-	-	-	-	72,4%	26,9%
20	Emeh et al. (2021) [19]		-	-	-	-	-	-	-	-	73,3%	26,7%

### Most known obstetric danger sign

**Fig. 2:**
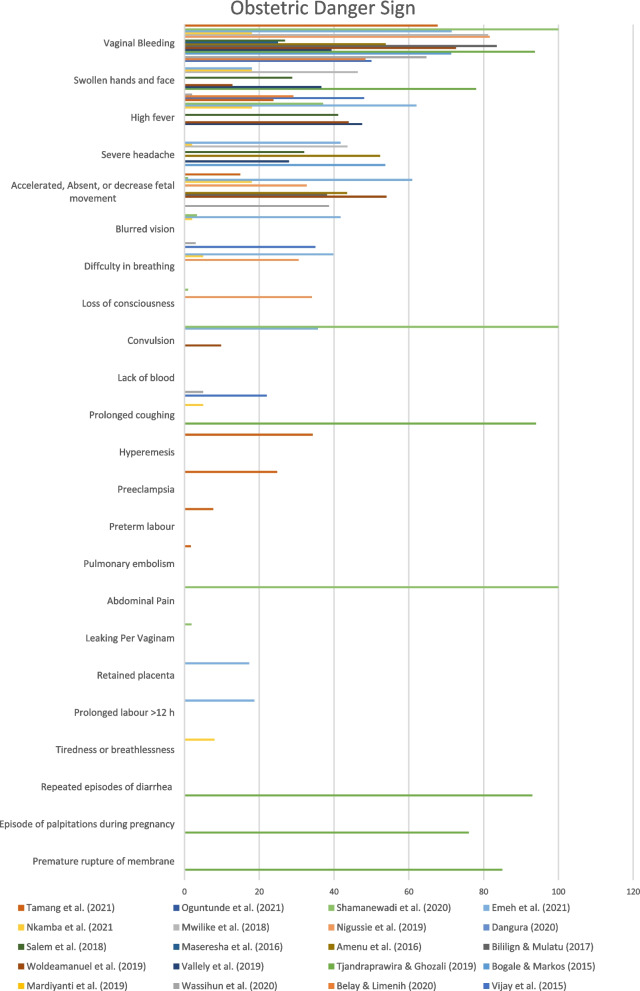
The chart of the most known obstetric danger sign

There were five danger signs of pregnancy that often occur, namely vaginal bleeding, swelling of the feet and hands, infection, premature rupture of membranes, and reduced fetal movement [1, 5–8, 13, 14, 16, 18–25, 31–33] (Fig. 2).

### Determinant of awareness

Factors related to the level of awareness of pregnant women about the danger signs of pregnancy (see Table 5), namely the first is Educational Status. Mothers with formal education have higher knowledge of the danger signs of pregnancy compared to illiterate mothers [24, 25, 31, 32]. This was in line with research conducted by Wassihun et al. [21] which said that respondents who have formal education are 6.01 times more likely to have good knowledge about the danger signs of pregnancy.

**Table 5:** Determinant of awareness

No	Determinant of danger sign’s awareness	Facts	Sources
1	Live in Urban area	• Live in urban area is more knowledgeable of the three (during pregnancy, childbirth, postpartum) • Women from Hiri district (peri-urban) are more knowledgeable compared with women from Asaro (rural) or Karkar (rural) – Papua New Guinea • Pregnant women live in the urban area are more knowledgeable • Mother who lives in the urban are more knowledgeable of the danger sign of postpartum	(Bogale & Markos, 2015), (Vallely et al., 2019), (Woldeamanuel et al., 2019), (Maseresha et al., 2016), (Dangura, 2020), (Shamanewadi et al., 2020) [4, 5, 16, 22, 23, 25]
2	Completed secondary school education	• A maternal educational level of formal secondary school or above is more knowledgeable of the three than the unable to read and write • Able to read and write is more knowledgeable of the three (during pregnancy, childbirth, postpartum) than unable to • Women with secondary and tertiary education are more knowledgeable compared with women having no education or primary education only • Mothers attended secondary education are more knowledgeable about the danger sign during pregnancy and postpartum period • Mother who finished more than secondary school are more knowledgeable • Have higher education are more knowledgeable • Attended secondary level and above are more knowledgeable of the danger sign of (1) childbirth • Higher level education is related to higher awareness	(Belay & Limenih, 2020), (Wassihun et al., 2020), (Bogale & Markos, 2015), (Vallely et al., 2019), (Woldeamanuel et al., 2019), (Bililign & Mulatu, 2017), (Amenu et al., 2016), (Salem et al., 2018), (Dangura, 2020), (Emeh et al., 2021) [7, 16, 19–23, 25, 31, 32]
3	Her husband completed at least secondary school education	• Mothers are more knowledgeable if her Husband finished more than secondary school	(Amenu et al., 2016) [7]
4	Had visit ANC at least 4 times in the last pregnancy	• Women who had an ANC follow-up visit in their last pregnancy were more likely to know three or than who did not have history of an ANC follow-up visit • Attended routine ANC is more knowledgeable than never attended • Visiting more antenatal care ≥ 4 make mothers more knowledgeable about the during pregnancy danger sign • Mothers who had ANC follow-up during last pregnancy are more knowledgeable • Pregnant women who visit the ANC are more knowledgeable • Mother initiates first ANC in the third trimester are more knowledgeable • The use of ANC during last pregnancy are more knowledgeable of the danger sign of childbirth and postpartum • Woman started the ANC at the first trimester is more aware • Woman attended ANC more often is more aware	(Belay & Limenih, 2020), (Wassihun et al., 2020), (Bogale & Markos, 2015), (Bililign & Mulatu, 2017), (Amenu et al., 2016), (Nigussie et al., 2019), (Dangura, 2020), (Emeh et al., 2021), (Oguntunde et al., 2021; Shamanewadi et al., 2020; Tamang et al., 2021) [5, 7, 8, 14, 18–23, 32]
5	Had bad obstetric experience such as complication	Women who had experienced a bad obstetric history (still birth and abortion) or experience complication is more knowledgeable of the three (during pregnancy, childbirth, postpartum)	(Belay & Limenih, 2020) [32]
16	Had obstetric health education especially in the ANC	• Women informed about obstetric danger signs during their last pregnancy is more knowledgeable of 1 and 2 than the non-informed • Exposed to danger information is more knowledgeable than not exposed • Women who received information at the antenatal clinic are more knowledgeable • Women receive health education are more knowledgeable • Mothers receive more information on the danger sign are more knowledgeable	(Belay & Limenih, 2020), (Mardiyanti et al., 2019), (Vallely et al., 2019), (Woldeamanuel et al., 2019), (Salem et al., 2018) [13, 16, 25, 31, 32]
7	Latest childbirth in the health facility	• Mothers who gave their last birth at a health institution is more likely to know the danger sign during pregnancy • Delivered in the health facility is more knowledgeable than delivered at home • Mothers last labour in the health facility are more knowledgeable about the during childbirth danger sign • Mother delivered the latest baby in the health facility are more knowledgeable • Mother delivered previous birth at health institution are more knowledgeable of the danger sign of postpartum • Mother delivered latest baby in the health institution are more knowledgeable of childbirth and postpartum	(Belay & Limenih, 2020), (Wassihun et al., 2020), (Bililign & Mulatu, 2017), (Amenu et al., 2016), (Dangura, 2020) [7, 20, 21, 23, 32]
8	Have a job	• Working as the government employee is more knowledgeable than housewives • Mothers work as private employees are more knowledgeable about the during pregnancy danger sign • Mothers who is Government employee and merchant are more knowledgeable	(Wassihun et al., 2020), (Bogale & Markos, 2015), (Bililign & Mulatu, 2017), (Nigussie et al., 2019) [8, 20–22]
9	Multiparous	• Multiparous woman is more knowledgeable than the primiparous • Women have more than 2 histories of pregnancy are more knowledgeable • Grand multipara mothers are more knowledgeable	(Mardiyanti et al., 2019), (Woldeamanuel et al., 2019), (Amenu et al., 2016) [7, 13, 25]
10	Better Knowledge	Have good knowledge is more knowledgeable than have less knowledge	(Mardiyanti et al., 2019), (Oguntunde et al., 2021) [13, 18]
11	Older than 35 years old	• Mother aged > 35 years old are more knowledgeable of postpartum • Older women are more knowledgeable • Woman aged above 46 years old are more aware	(Dangura, 2020), (Mwilike, Nalwadda, et al., 2018), (Emeh et al., 2021) [19, 23, 33]
12	Bad awareness on mother less than 24 years old	There was no significant association age (under 24 years compared with age 25 and over)	(Vallely et al., 2019) [16]
13	Close distance to health facility around 20 – 1 h trip	• The distance between house and health facility < 20 min on foot are more knowledgeable • Distance to health facility < 1 h are more knowledgeable of childbirth	(Woldeamanuel et al., 2019), (Dangura, 2020) [23, 25]
14	Family income > 27 – 40 USD	• Family monthly income ≥ 1500 ETB (Ethiopian Birr) ≈ 27,73 USD are more knowledgeable • Mother who has monthly income of 40 USD are more knowledgeable of (2) postpartum	(Amenu et al., 2016), (Dangura, 2020) [7, 23]
15	Currently pregnant	Women who is pregnant > 5 times are more knowledgeable Mothers live with higher income are more knowledgeable	(Maseresha et al., 2016), (Salem et al., 2018) [4, 31]
16	Participated in health survey	Participate in the PANDA mHealth project are more knowledgeable	(Salem et al., 2018) [31]
17	Autonomy for women by the family	Women authorized to decide to have medical care by the family are more knowledgeable	(Nigussie et al., 2019) [8]
18	Active source of information such as TV and Radio	Have functioning radio/ tv in the house are more knowledgeable of childbirth and postpartum	(Dangura, 2020) [23]

The second is pregnant experience, the results of research conducted by Mwilike et al. [27, 33] showed that there was a strong relationship between pregnant experience and knowledge of the danger signs of pregnancy. This was because women who have more pregnant experience have often received information, thus increasing their knowledge.

The third is ANC visit, the number of ANC visit can significantly affect knowledge about the danger signs of pregnancy. Respondents who had antenatal care visits were 1.26 times more likely to have good knowledge of the danger signs of pregnancy [21, 31, 32]. This was in line with a study conducted by Vallely et al [16] which stated that women who received information during ANC visit were almost eight times more likely to know the danger signs of pregnancy compared to women who did not.

The last is place of delivery, mother who gave birth in health services were 5.7 times more likely to have knowledge of the danger signs of pregnancy than respondents who gave birth at home [21]. This was in line with research conducted by Belay & Limenih [32] stated that mothers who the latest child delivery was in a health care facility were more likely to know three or more obstetric danger signs.

Furthermore, awareness of the obstetric danger signs was an important alarm to receive appropriate and timely referrals for care during pregnancy, childbirth, and postpartum [22]. Most maternal deaths can be avoided, if the mother and family are aware of the obstetric danger signs. Dangura [23] stated that increasing maternal awareness about the danger signs of pregnancy can increase early detection of problems and reduce delays in deciding to seek care.

## Discussion

The majority of studies do not report the awareness using direct measurement, but many studies that aim to examine awareness use knowledge as the criteria [1, 4, 7, 8, 14, 16–25, 31–33]. Surprisingly the pregnant women’s low awareness of obstetric danger sing in the developing country is partly incorrect. The studies in developing countries shown mother to have good level of awareness, proven by the ability to recall the knowledge of obstetric danger sign [1, 7, 19, 25, 31, 32].

Many developing countries have addressed the gap of low level of obstetric danger sign awareness by increase ANC facilities, and improve the quality, but the evidence about the adequate resources in the facility is a question that beyond the scope of this review. The problem that increased the MMR in developing countries is not lay in the process of ANC [34–36]. Somehow, this review found that the barriers for women to seek medical care promptly when the obstetric danger sign is present could be the culprit for the high number of MMR [4, 5, 14, 19, 21]. The barriers such as the family issue on taking action for the aware mother when the danger sign is appeared, practically the most neglected factors during the ANC visit [14, 37, 38]. The ANC visit focusing in the pregnancy health, improve the knowledge, and administer medicine or vitamin to improve the pregnancy health [31].

Surprisingly education and economic status does not play any major role in the mother awareness [8, 18]. Despite, some research state that the better the education and the economic status would make mother retain information better [5, 14, 16]. However, the case in obstetric danger sign awareness is different. It is not about the internal characteristics of the mother, but more of a social support and the health education that the mother may obtain during ANC visit [14, 37, 38]. Programs for pregnant and giving birth women including their services are provided free of charge by many developing countries from national or international support [1, 14, 18, 22].

The recommendation for clinical practice to improve mother awareness about the obstetric danger sign is to improve the effectiveness of the health education during the ANC [15, 19, 20, 31]. Mother who is more experienced in pregnancy have better knowledge about obstetric danger sign and the more she would visit the ANC [6, 19, 23]. There should be a clear strategy to transfer all the obstetric danger sign information, especially to the mother with high risk factors [1, 19, 20].

An MCH handbook (Maternal and Child Health) is a good media to for information transfer [1, 14]. This book is an integral of medical record. A medical record only available for the health care, yet the MCH handbook is available for both parties, mother and the health care. Even the family could have access to the book. This could inform what is found during the ANC to the family. This would improve the mother’s knowledge of danger sign, including the family [1, 14, 27]. Mother in developing country reported that awareness begins from their household. In the form of partner or family support of their pregnancy [17, 18, 39]. The MCH handbook would facilitate the information transfer to the family and especially the father, the head of the family, or the dominant elders. This can support the mother to seek medical care for dangerous signs and symptoms appear [1, 14, 27]. Furthermore, an application to support ANC such as the mhealth (mobile health) in Madagascar could be an alternative. So that information related to the health of pregnant women can be more extensive and increase the involvement of younger pregnant women [5, 31].

The ANC is the perfect place to deliver health education, but the efficiency of the ANC might be hindered by the barriers on mother seeking medical help [6, 7, 19, 20]. The assessment about the barrier of health seeking should be part of the ANC program. Mother needs to be encouraged to express the barrier of health seeking [19, 23]. The nurse or other health care staff should help the mother to find the solutions for particular barrier. Nurse care plan includes family intervention. Nurse need to treat the family problem related to the health seeking of pregnant mother [33, 40]. This review suggests developing country should start to include assessment of barrier of health seeking and use an MCH handbook into the ANC program.

## Conclusion

The empirical data of pregnant mother awareness about the obstetric danger sign in developing country is low to medium. Only a handful of developing country have fair awareness, in which related to determinant of awareness. It is better educational status, more pregnant experience, more ANC visit, place of delivery in the health care facility. The recommended effective strategy to reinforce women awareness is by waive the barrier of health seeking, especially in the household level. The assessment of barrier of health seeking should be part of ANC program. The barrier of health seeking is unique in the developing country. Mostly it is related to the family support, i.e. the husband and the elderly. Additionally, use MCH handbook or mobile application to record the ANC visit is also beneficial to communicate with the family.

## Data Availability

The datasets used and/or analysed during the current study available from the corresponding author on reasonable request.

## Abbreviations

MMR: Maternal Mortality Rate
IDHS: Indonesian Demographic and Health Survey
SDGs: Sustainable Development Goals
PRISMA-ScR: Preferred Reporting Items for Systematic reviews and Meta-Analyses extension for Scoping Reviews
PICO: Patient/ Problem, Interest/ Intervention, Comparison/ Control/ Comparator, Outcome
CASP: Critical Appraisal Skills Programme
ANC: Antenatal Care
WHO: World Health Organization
mHealrh: Mobile Health
MCH: Mother and Child Health
